# Supplementary material for: Role and mechanism of NCAPD3 in promoting malignant behaviors in gastric cancer
Source: Front Pharmacol. 2024 Apr 22;15:1341039. doi: 10.3389/fphar.2024.1341039 (PMC11070777; doi:10.3389/fphar.2024.1341039)
Supplement: Supplementary file 11 [file DataSheet2.ZIP › GSEA/Canonical pathways/my_analysis.Gsea.1599462267220/REACTOME_TOLL_LIKE_RECEPTOR_CASCADES.html]

Details for gene set REACTOME\_TOLL\_LIKE\_RECEPTOR\_CASCADES[GSEA]

|  || Dataset | filtered\_dataset.sample\_info.cls#WT\_versus\_NCAPD3\_MUT |
| Phenotype | sample\_info.cls#WT\_versus\_NCAPD3\_MUT |
| Upregulated in class | WT |
| GeneSet | REACTOME\_TOLL\_LIKE\_RECEPTOR\_CASCADES |
| Enrichment Score (ES) | 0.289542 |
| Normalized Enrichment Score (NES) | 1.1069509 |
| Nominal p-value | 0.33730835 |
| FDR q-value | 0.71728224 |
| FWER p-Value | 1.0 |
Table: GSEA Results Summary

  

Fig 1: Enrichment plot: REACTOME\_TOLL\_LIKE\_RECEPTOR\_CASCADES      
 Profile of the Running ES Score & Positions of GeneSet Members on the Rank Ordered List

  

| SYMBOL | TITLE | RANK IN GENE LIST | RANK METRIC SCORE | RUNNING ES | CORE ENRICHMENT || 1 | 2243 | FGA | 18 | 1.039 | 0.1127 | Yes |
| 2 | 3654 | IRAK1 | 69 | 0.856 | 0.1804 | Yes |
| 3 | 51135 | IRAK4 | 245 | 0.635 | 0.1318 | Yes |
| 4 | 353376 | TICAM2 | 271 | 0.617 | 0.1885 | Yes |
| 5 | 2244 | FGB | 321 | 0.582 | 0.2237 | Yes |
| 6 | 8454 | CUL1 | 492 | 0.477 | 0.1596 | Yes |
| 7 | 8737 | RIPK1 | 493 | 0.477 | 0.2173 | Yes |
| 8 | 7099 | TLR4 | 541 | 0.451 | 0.2381 | Yes |
| 9 | 7334 | UBE2N | 546 | 0.449 | 0.2895 | Yes |
| 10 | 6197 | RPS6KA3 | 643 | 0.403 | 0.2695 | No |
| 11 | 8772 | FADD | 742 | 0.355 | 0.2422 | No |
| 12 | 3146 | HMGB1 | 831 | 0.295 | 0.2149 | No |
| 13 | 3665 | IRF7 | 956 | -0.375 | 0.1713 | No |
| 14 | 330 | BIRC3 | 1224 | -0.600 | 0.0526 | No |
| 15 | 8651 | SOCS1 | 1272 | -0.661 | 0.0989 | No |
Table: GSEA details [plain text format]

  

Fig 2: REACTOME\_TOLL\_LIKE\_RECEPTOR\_CASCADES      
 Blue-Pink O' Gram in the Space of the Analyzed GeneSet

  

Fig 3: REACTOME\_TOLL\_LIKE\_RECEPTOR\_CASCADES: Random ES distribution      
 Gene set null distribution of ES for **REACTOME\_TOLL\_LIKE\_RECEPTOR\_CASCADES**
